# (*Z*)-3-(9-Anthryl)-1-(4-chloro­phen­yl)-2-(4-nitro-1*H*-imidazol-1-yl)prop-2-en-1-one

**DOI:** 10.1107/S1600536809014676

**Published:** 2009-04-25

**Authors:** Guang-zhou Wang, Yi-hui Lu, Cheng-he Zhou, Yi-yi Zhang

**Affiliations:** a School of Chemistry and Chemical Engineering, Southwest University, Chongqing 400715, People’s Republic of China

## Abstract

In the title compound, C_26_H_16_ClN_3_O_3_, the dihedral angle between the anthracene mean plane and imidazole ring is 64.75 (2)°. In the crystal, π–π inter­actions between anthracene fragments lead to the formation of stacks of mol­ecules propagating in [100]. The short distance between the carbonyl groups of symmetry-related molecules [C⋯O = 2.985 (2) Å] indicates the existence of dipole–dipole inter­actions. The crystal packing also exhibits short inter­molecular contacts between the nitro groups and Cl atoms [Cl⋯O = 3.181 (2) Å].

## Related literature

For general background, see: Corrêa *et al.* (2001 ▶); Daskiewicz *et al.* (2005 ▶); Sivakumar *et al.* (2009 ▶); Vogel *et al.* (2008 ▶). The synthesis was described by Erhardt *et al.* (1985 ▶).
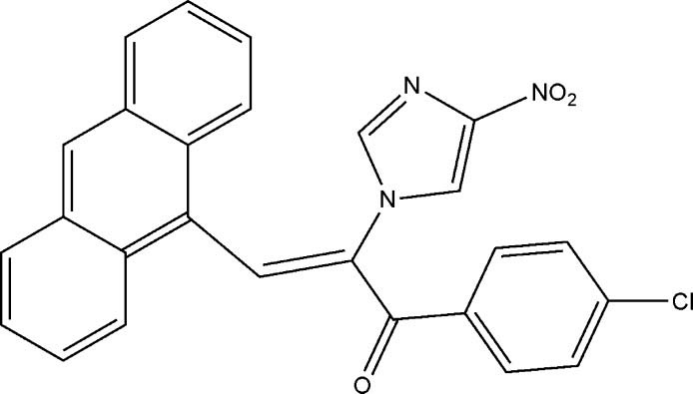


## Experimental

### 

#### Crystal data

C_26_H_16_ClN_3_O_3_
*M* *_r_* = 453.87
Triclinic, 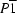

*a* = 8.0511 (9) Å
*b* = 11.0406 (12) Å
*c* = 12.9274 (14) Å
α = 76.065 (2)°
β = 85.974 (2)°
γ = 71.258 (2)°
*V* = 1056.1 (2) Å^3^
*Z* = 2
Mo *K*α radiation
μ = 0.22 mm^−1^
*T* = 292 K
0.16 × 0.12 × 0.10 mm

#### Data collection

Bruker SMART APEX CCD area-detector diffractometer
Absorption correction: multi-scan (*SADABS*; Sheldrick, 1997 ▶) *T*_min_ = 0.956, *T*_max_ = 0.979
6168 measured reflections
4070 independent reflections
3354 reflections with *I* > 2σ(*I*)
*R*_int_ = 0.014

#### Refinement

*R*[*F* ^2^ > 2σ(*F* ^2^)] = 0.044
*wR*(*F* ^2^) = 0.113
*S* = 1.04
4070 reflections
299 parameters
H-atom parameters constrained
Δρ_max_ = 0.56 e Å^−3^
Δρ_min_ = −0.50 e Å^−3^

### 

Data collection: *SMART* (Bruker, 2001 ▶); cell refinement: *SAINT* (Bruker, 2001 ▶); data reduction: *SAINT*; program(s) used to solve structure: *SHELXS97* (Sheldrick, 2008 ▶); program(s) used to refine structure: *SHELXL97* (Sheldrick, 2008 ▶); molecular graphics: *PLATON* (Spek, 2009 ▶); software used to prepare material for publication: *PLATON*.

## Supplementary Material

Crystal structure: contains datablocks global, I. DOI: 10.1107/S1600536809014676/cv2548sup1.cif

Structure factors: contains datablocks I. DOI: 10.1107/S1600536809014676/cv2548Isup2.hkl

Additional supplementary materials:  crystallographic information; 3D view; checkCIF report

## Figures and Tables

**Table 1:** Selected interatomic distances (Å)

C17⋯O1^i^	2.985 (2)
Cl1⋯O3^ii^	3.181 (3)
*Cg*1⋯*Cg*2^iii^	3.746 (7)
*Cg*2⋯*Cg*2^iv^	3.863 (8)

Symmetry codes: (i) 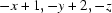; (ii) 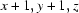; (iii) 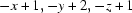; (iv) 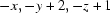. *Cg*1 and *Cg*2 are the centroids of atoms C1/C2/C14/C7–C9 and C2–C7, respectively.

**Fig. 1.**
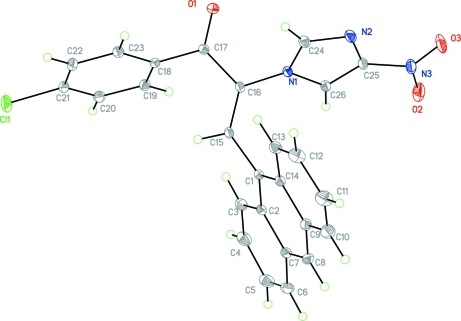
The molecular structure of (I) showing the atomic numbering and 30% probability displacement ellipsoids.

## supplementary crystallographic information

### Comment

Chalcones or 1,3-diaryl-2-propen-1-ones are natural or synthetic compounds belonging to the flavonoid family (Corrêa *et al.*, 2001). They exhibit different kinds of biological activities, such as antimicrobial, anticancer, antiviral, ant-malarial, anti-inflammatory activities (Daskiewicz *et al.*, 2005; Vogel *et al.*, 2008; Sivakumar *et al.*, 2009). Hence, chalcones are considered as a class of important therapeutic potentials. The title compound, (I), is part of our effort in order to contribute this research, and we report its crystal structure here.

In (I) (Fig. 1), the dihedral angle between the anthracene and imidazole rings is 64.75 (2)° and the nitroimidazole is slightly twisted away from the 4-chlorophenyl ring with a dihedral angle of 11.55 (2)°. In the crystal, the π-π interactions between the anthracene fragments (Table 1) lead to formation of stacks of the molecules propagated in direction [100]. The short distance between the carbonyl groups [C17···O1(1-x, 2-y, -z) 2.985 (2) Å] (Table 1) reveals an existence of dipole-dipole interactions. The crystal packing also exhibits short intermolecular contacts between the nitro groups and chlorine atoms [Cl···O 3.181 (2) Å] (Table 1).

### Experimental

Compound (I) was synthesized according to the procedure of Erhardt *et al.* (1985). A crystal of (I) suitable for X-ray analysis was grown from a mixture solution of chloroform and acetone by slow evaporation at room temperature.

### Refinement

All the hydrogen atoms were placed at their geometrical positions with C—H = 0.93Å and *U*_iso_(H) = 1.2*U*_eq_(C).

### Crystal data

**Table:**

C_26_H_16_ClN_3_O_3_	*Z* = 2
*M**_r_* = 453.87	*F*(000) = 468
Triclinic, *P*1	*D*_x_ = 1.427 Mg m^−^^3^
Hall symbol: -P 1	Mo *K*α radiation, λ = 0.71073 Å
*a* = 8.0511 (9) Å	Cell parameters from 2837 reflections
*b* = 11.0406 (12) Å	θ = 1.4–25.3°
*c* = 12.9274 (14) Å	µ = 0.22 mm^−^^1^
α = 76.065 (2)°	*T* = 292 K
β = 85.974 (2)°	Block, orange
γ = 71.258 (2)°	0.16 × 0.12 × 0.10 mm
*V* = 1056.1 (2) Å^3^	

### Data collection

**Table:**

Bruker SMART APEX CCD area-detector diffractometer	4070 independent reflections
Radiation source: fine focus sealed Siemens Mo tube	3354 reflections with *I* > 2σ(*I*)
graphite	*R*_int_ = 0.014
0.3° wide ω exposures scans	θ_max_ = 26.0°, θ_min_ = 2.0°
Absorption correction: multi-scan (*SADABS*; Sheldrick, 1997)	*h* = −9→9
*T*_min_ = 0.956, *T*_max_ = 0.979	*k* = −13→10
6168 measured reflections	*l* = −15→15

### Refinement

**Table:**

Refinement on *F*^2^	Secondary atom site location: difference Fourier map
Least-squares matrix: full	Hydrogen site location: inferred from neighbouring sites
*R*[*F*^2^ > 2σ(*F*^2^)] = 0.044	H-atom parameters constrained
*wR*(*F*^2^) = 0.113	*w* = 1/[σ^2^(*F*_o_^2^) + (0.0441*P*)^2^ + 0.4068*P*] where *P* = (*F*_o_^2^ + 2*F*_c_^2^)/3
*S* = 1.04	(Δ/σ)_max_ = 0.001
4070 reflections	Δρ_max_ = 0.56 e Å^−^^3^
299 parameters	Δρ_min_ = −0.50 e Å^−^^3^
0 restraints	Extinction correction: *SHELXL97* (Sheldrick, 2008), Fc^*^=kFc[1+0.001xFc^2^λ^3^/sin(2θ)]^-1/4^
Primary atom site location: structure-invariant direct methods	Extinction coefficient: 0.138 (5)

### Special details

**Table:**

Geometry. All e.s.d.'s (except the e.s.d. in the dihedral angle between two l.s. planes) are estimated using the full covariance matrix. The cell e.s.d.'s are taken into account individually in the estimation of e.s.d.'s in distances, angles and torsion angles; correlations between e.s.d.'s in cell parameters are only used when they are defined by crystal symmetry. An approximate (isotropic) treatment of cell e.s.d.'s is used for estimating e.s.d.'s involving l.s. planes.
Refinement. Refinement of *F*^2^ against ALL reflections. The weighted *R*-factor *wR* and goodness of fit *S* are based on *F*^2^, conventional *R*-factors *R* are based on *F*, with *F* set to zero for negative *F*^2^. The threshold expression of *F*^2^ > σ(*F*^2^) is used only for calculating *R*-factors(gt) *etc*. and is not relevant to the choice of reflections for refinement. *R*-factors based on *F*^2^ are statistically about twice as large as those based on *F*, and *R*- factors based on ALL data will be even larger.

### Fractional atomic coordinates and isotropic or equivalent isotropic displacement parameters (Å^2^)

**Table:**

	*x*	*y*	*z*	*U*_iso_*/*U*_eq_	
C1	0.4503 (2)	0.91044 (16)	0.38505 (12)	0.0381 (4)	
C2	0.3150 (2)	0.99845 (18)	0.43057 (13)	0.0427 (4)	
C3	0.2324 (3)	1.13103 (19)	0.37653 (16)	0.0532 (5)	
H3	0.2673	1.1618	0.3078	0.064*	
C4	0.1039 (3)	1.2135 (2)	0.4233 (2)	0.0690 (6)	
H4	0.0521	1.2999	0.3863	0.083*	
C5	0.0482 (3)	1.1702 (3)	0.5267 (2)	0.0758 (7)	
H5	−0.0389	1.2284	0.5581	0.091*	
C6	0.1195 (3)	1.0455 (3)	0.58072 (18)	0.0671 (6)	
H6	0.0806	1.0181	0.6491	0.080*	
C7	0.2544 (2)	0.9537 (2)	0.53486 (14)	0.0507 (5)	
C8	0.3284 (3)	0.8240 (2)	0.58804 (14)	0.0570 (5)	
H8	0.2870	0.7948	0.6553	0.068*	
C9	0.4618 (3)	0.7360 (2)	0.54475 (14)	0.0528 (5)	
C10	0.5404 (4)	0.6035 (2)	0.60138 (18)	0.0751 (7)	
H10	0.4981	0.5739	0.6682	0.090*	
C11	0.6738 (4)	0.5211 (2)	0.5604 (2)	0.0864 (8)	
H11	0.7210	0.4345	0.5983	0.104*	
C12	0.7438 (4)	0.5643 (2)	0.45999 (19)	0.0730 (7)	
H12	0.8385	0.5065	0.4332	0.088*	
C13	0.6736 (3)	0.68926 (18)	0.40237 (15)	0.0516 (5)	
H13	0.7218	0.7164	0.3368	0.062*	
C14	0.5276 (2)	0.77936 (17)	0.44076 (13)	0.0428 (4)	
C15	0.5225 (2)	0.96316 (15)	0.28220 (12)	0.0370 (4)	
H15	0.5605	1.0348	0.2804	0.044*	
C16	0.5410 (2)	0.92308 (15)	0.19171 (12)	0.0351 (4)	
C17	0.6425 (2)	0.97302 (15)	0.10011 (12)	0.0364 (4)	
C18	0.6982 (2)	1.08892 (16)	0.10310 (12)	0.0372 (4)	
C19	0.5813 (2)	1.21059 (17)	0.10958 (14)	0.0442 (4)	
H19	0.4615	1.2227	0.1122	0.053*	
C20	0.6414 (3)	1.31410 (18)	0.11219 (15)	0.0503 (5)	
H20	0.5631	1.3961	0.1153	0.060*	
C21	0.8197 (3)	1.29305 (19)	0.11011 (15)	0.0532 (5)	
C22	0.9379 (3)	1.1743 (2)	0.10195 (16)	0.0563 (5)	
H22	1.0576	1.1626	0.0995	0.068*	
C23	0.8762 (2)	1.07254 (18)	0.09746 (15)	0.0474 (4)	
H23	0.9550	0.9922	0.0906	0.057*	
C24	0.5604 (3)	0.70649 (17)	0.15237 (15)	0.0488 (4)	
H24	0.6791	0.6812	0.1360	0.059*	
C25	0.2993 (3)	0.70981 (18)	0.17933 (14)	0.0480 (4)	
C26	0.3024 (2)	0.82478 (17)	0.19785 (13)	0.0425 (4)	
H26	0.2098	0.8902	0.2183	0.051*	
Cl1	0.90255 (10)	1.41713 (6)	0.11994 (6)	0.0886 (3)	
N1	0.47212 (18)	0.82266 (13)	0.17975 (10)	0.0378 (3)	
N2	0.4588 (2)	0.63534 (15)	0.15188 (13)	0.0554 (4)	
N3	0.1473 (3)	0.6670 (2)	0.18554 (14)	0.0685 (5)	
O1	0.68863 (17)	0.91581 (12)	0.02841 (10)	0.0486 (3)	
O2	0.0083 (3)	0.7406 (2)	0.21193 (17)	0.0925 (6)	
O3	0.1648 (3)	0.5612 (2)	0.16539 (18)	0.1061 (7)	

### Atomic displacement parameters (Å^2^)

**Table:**

	*U*^11^	*U*^22^	*U*^33^	*U*^12^	*U*^13^	*U*^23^
C1	0.0429 (9)	0.0474 (9)	0.0326 (8)	−0.0239 (7)	−0.0008 (7)	−0.0116 (7)
C2	0.0408 (9)	0.0578 (11)	0.0399 (9)	−0.0234 (8)	0.0005 (7)	−0.0200 (8)
C3	0.0511 (11)	0.0579 (12)	0.0554 (11)	−0.0168 (9)	−0.0008 (9)	−0.0223 (9)
C4	0.0561 (13)	0.0703 (14)	0.0842 (16)	−0.0094 (11)	−0.0040 (11)	−0.0377 (13)
C5	0.0495 (13)	0.108 (2)	0.0832 (17)	−0.0176 (13)	0.0071 (11)	−0.0576 (16)
C6	0.0482 (12)	0.119 (2)	0.0535 (12)	−0.0369 (13)	0.0112 (9)	−0.0440 (13)
C7	0.0441 (10)	0.0823 (14)	0.0407 (9)	−0.0335 (10)	0.0028 (8)	−0.0241 (10)
C8	0.0605 (12)	0.0901 (16)	0.0345 (9)	−0.0445 (12)	0.0046 (8)	−0.0135 (10)
C9	0.0681 (13)	0.0633 (12)	0.0366 (9)	−0.0377 (10)	−0.0063 (8)	−0.0040 (8)
C10	0.113 (2)	0.0690 (15)	0.0459 (12)	−0.0430 (15)	−0.0093 (12)	0.0035 (11)
C11	0.137 (3)	0.0527 (13)	0.0578 (14)	−0.0232 (15)	−0.0204 (15)	0.0063 (11)
C12	0.0934 (18)	0.0528 (12)	0.0627 (14)	−0.0081 (12)	−0.0162 (12)	−0.0099 (10)
C13	0.0619 (12)	0.0490 (10)	0.0441 (10)	−0.0177 (9)	−0.0071 (9)	−0.0085 (8)
C14	0.0505 (10)	0.0486 (10)	0.0365 (9)	−0.0249 (8)	−0.0043 (7)	−0.0089 (7)
C15	0.0408 (9)	0.0366 (8)	0.0385 (8)	−0.0177 (7)	−0.0019 (7)	−0.0091 (7)
C16	0.0384 (9)	0.0322 (8)	0.0376 (8)	−0.0142 (6)	−0.0030 (6)	−0.0083 (6)
C17	0.0361 (8)	0.0373 (8)	0.0358 (8)	−0.0104 (7)	−0.0018 (6)	−0.0092 (7)
C18	0.0439 (9)	0.0390 (8)	0.0318 (8)	−0.0178 (7)	0.0038 (6)	−0.0080 (6)
C19	0.0456 (10)	0.0416 (9)	0.0458 (9)	−0.0154 (8)	0.0034 (7)	−0.0092 (7)
C20	0.0647 (12)	0.0386 (9)	0.0493 (10)	−0.0194 (8)	0.0072 (9)	−0.0107 (8)
C21	0.0713 (13)	0.0516 (11)	0.0508 (11)	−0.0378 (10)	0.0146 (9)	−0.0169 (9)
C22	0.0508 (11)	0.0676 (13)	0.0648 (12)	−0.0333 (10)	0.0149 (9)	−0.0256 (10)
C23	0.0452 (10)	0.0499 (10)	0.0526 (10)	−0.0186 (8)	0.0091 (8)	−0.0195 (8)
C24	0.0616 (12)	0.0385 (9)	0.0507 (10)	−0.0185 (8)	0.0026 (8)	−0.0152 (8)
C25	0.0647 (12)	0.0497 (10)	0.0391 (9)	−0.0343 (9)	−0.0065 (8)	−0.0038 (8)
C26	0.0461 (10)	0.0467 (9)	0.0402 (9)	−0.0221 (8)	−0.0022 (7)	−0.0089 (7)
Cl1	0.1041 (5)	0.0745 (4)	0.1183 (6)	−0.0626 (4)	0.0236 (4)	−0.0392 (4)
N1	0.0449 (8)	0.0364 (7)	0.0372 (7)	−0.0181 (6)	−0.0005 (6)	−0.0107 (6)
N2	0.0803 (12)	0.0435 (9)	0.0524 (9)	−0.0313 (8)	−0.0009 (8)	−0.0133 (7)
N3	0.0902 (15)	0.0785 (13)	0.0566 (10)	−0.0601 (12)	−0.0108 (10)	−0.0030 (9)
O1	0.0552 (8)	0.0524 (7)	0.0465 (7)	−0.0217 (6)	0.0111 (6)	−0.0232 (6)
O2	0.0715 (12)	0.1073 (15)	0.1141 (15)	−0.0560 (11)	0.0001 (11)	−0.0155 (12)
O3	0.1425 (18)	0.1018 (14)	0.1193 (16)	−0.0927 (14)	−0.0026 (13)	−0.0342 (12)

### Geometric parameters (Å, °)

**Table:**

C1—C2	1.408 (2)	C15—H15	0.9300
C1—C14	1.408 (2)	C16—N1	1.4318 (19)
C1—C15	1.477 (2)	C16—C17	1.488 (2)
C2—C3	1.421 (3)	C17—O1	1.2162 (19)
C2—C7	1.429 (2)	C17—C18	1.495 (2)
C3—C4	1.355 (3)	C18—C23	1.384 (2)
C3—H3	0.9300	C18—C19	1.389 (2)
C4—C5	1.402 (4)	C19—C20	1.384 (2)
C4—H4	0.9300	C19—H19	0.9300
C5—C6	1.341 (4)	C20—C21	1.378 (3)
C5—H5	0.9300	C20—H20	0.9300
C6—C7	1.432 (3)	C21—C22	1.374 (3)
C6—H6	0.9300	C21—Cl1	1.7382 (18)
C7—C8	1.384 (3)	C22—C23	1.380 (3)
C8—C9	1.385 (3)	C22—H22	0.9300
C8—H8	0.9300	C23—H23	0.9300
C9—C10	1.425 (3)	C24—N2	1.305 (2)
C9—C14	1.439 (2)	C24—N1	1.369 (2)
C10—C11	1.336 (4)	C24—H24	0.9300
C10—H10	0.9300	C25—C26	1.355 (2)
C11—C12	1.415 (4)	C25—N2	1.360 (3)
C11—H11	0.9300	C25—N3	1.437 (3)
C12—C13	1.357 (3)	C26—N1	1.364 (2)
C12—H12	0.9300	C26—H26	0.9300
C13—C14	1.422 (3)	N3—O3	1.219 (3)
C13—H13	0.9300	N3—O2	1.235 (3)
C15—C16	1.330 (2)		
			
C17···O1^i^	2.985 (2)	Cg1···Cg2^iii^	3.746 (7)
Cl1···O3^ii^	3.181 (3)	Cg2···Cg2^iv^	3.863 (8)
			
C2—C1—C14	121.15 (15)	C16—C15—H15	115.3
C2—C1—C15	117.97 (15)	C1—C15—H15	115.3
C14—C1—C15	120.54 (15)	C15—C16—N1	121.23 (14)
C1—C2—C3	122.84 (16)	C15—C16—C17	122.54 (14)
C1—C2—C7	119.41 (17)	N1—C16—C17	116.12 (13)
C3—C2—C7	117.74 (17)	O1—C17—C16	120.77 (14)
C4—C3—C2	121.3 (2)	O1—C17—C18	120.97 (14)
C4—C3—H3	119.4	C16—C17—C18	118.09 (13)
C2—C3—H3	119.4	C23—C18—C19	119.23 (15)
C3—C4—C5	120.8 (2)	C23—C18—C17	117.30 (15)
C3—C4—H4	119.6	C19—C18—C17	123.47 (15)
C5—C4—H4	119.6	C20—C19—C18	120.67 (17)
C6—C5—C4	120.4 (2)	C20—C19—H19	119.7
C6—C5—H5	119.8	C18—C19—H19	119.7
C4—C5—H5	119.8	C21—C20—C19	118.55 (17)
C5—C6—C7	121.2 (2)	C21—C20—H20	120.7
C5—C6—H6	119.4	C19—C20—H20	120.7
C7—C6—H6	119.4	C22—C21—C20	121.89 (17)
C8—C7—C2	119.04 (17)	C22—C21—Cl1	117.65 (16)
C8—C7—C6	122.40 (19)	C20—C21—Cl1	120.45 (15)
C2—C7—C6	118.6 (2)	C21—C22—C23	118.98 (18)
C7—C8—C9	122.41 (17)	C21—C22—H22	120.5
C7—C8—H8	118.8	C23—C22—H22	120.5
C9—C8—H8	118.8	C22—C23—C18	120.63 (17)
C8—C9—C10	121.97 (19)	C22—C23—H23	119.7
C8—C9—C14	119.60 (18)	C18—C23—H23	119.7
C10—C9—C14	118.4 (2)	N2—C24—N1	112.21 (18)
C11—C10—C9	121.4 (2)	N2—C24—H24	123.9
C11—C10—H10	119.3	N1—C24—H24	123.9
C9—C10—H10	119.3	C26—C25—N2	112.81 (16)
C10—C11—C12	120.6 (2)	C26—C25—N3	125.7 (2)
C10—C11—H11	119.7	N2—C25—N3	121.48 (18)
C12—C11—H11	119.7	C25—C26—N1	104.26 (16)
C13—C12—C11	120.5 (2)	C25—C26—H26	127.9
C13—C12—H12	119.7	N1—C26—H26	127.9
C11—C12—H12	119.7	C26—N1—C24	106.95 (14)
C12—C13—C14	121.0 (2)	C26—N1—C16	125.02 (14)
C12—C13—H13	119.5	C24—N1—C16	128.00 (15)
C14—C13—H13	119.5	C24—N2—C25	103.77 (15)
C1—C14—C13	123.55 (16)	O3—N3—O2	124.9 (2)
C1—C14—C9	118.36 (17)	O3—N3—C25	118.1 (2)
C13—C14—C9	118.00 (17)	O2—N3—C25	116.95 (19)
C16—C15—C1	129.36 (14)		
			
C14—C1—C2—C3	−179.34 (15)	C1—C15—C16—C17	−169.86 (16)
C15—C1—C2—C3	7.3 (2)	C15—C16—C17—O1	164.24 (16)
C14—C1—C2—C7	−0.4 (2)	N1—C16—C17—O1	−12.1 (2)
C15—C1—C2—C7	−173.69 (14)	C15—C16—C17—C18	−11.0 (2)
C1—C2—C3—C4	−179.21 (18)	N1—C16—C17—C18	172.64 (13)
C7—C2—C3—C4	1.8 (3)	O1—C17—C18—C23	−54.0 (2)
C2—C3—C4—C5	−0.1 (3)	C16—C17—C18—C23	121.27 (17)
C3—C4—C5—C6	−1.0 (3)	O1—C17—C18—C19	125.09 (18)
C4—C5—C6—C7	0.4 (3)	C16—C17—C18—C19	−59.7 (2)
C1—C2—C7—C8	−1.1 (2)	C23—C18—C19—C20	−1.1 (3)
C3—C2—C7—C8	177.93 (16)	C17—C18—C19—C20	179.92 (15)
C1—C2—C7—C6	178.61 (15)	C18—C19—C20—C21	−1.1 (3)
C3—C2—C7—C6	−2.3 (2)	C19—C20—C21—C22	2.2 (3)
C5—C6—C7—C8	−178.96 (19)	C19—C20—C21—Cl1	−176.77 (14)
C5—C6—C7—C2	1.3 (3)	C20—C21—C22—C23	−1.1 (3)
C2—C7—C8—C9	1.3 (3)	Cl1—C21—C22—C23	177.95 (15)
C6—C7—C8—C9	−178.43 (17)	C21—C22—C23—C18	−1.2 (3)
C7—C8—C9—C10	178.23 (19)	C19—C18—C23—C22	2.2 (3)
C7—C8—C9—C14	0.0 (3)	C17—C18—C23—C22	−178.67 (16)
C8—C9—C10—C11	−177.3 (2)	N2—C25—C26—N1	0.8 (2)
C14—C9—C10—C11	0.9 (3)	N3—C25—C26—N1	−178.49 (16)
C9—C10—C11—C12	1.5 (4)	C25—C26—N1—C24	−0.45 (18)
C10—C11—C12—C13	−1.7 (4)	C25—C26—N1—C16	−178.48 (14)
C11—C12—C13—C14	−0.6 (3)	N2—C24—N1—C26	0.0 (2)
C2—C1—C14—C13	−174.85 (16)	N2—C24—N1—C16	177.96 (15)
C15—C1—C14—C13	−1.7 (2)	C15—C16—N1—C26	53.9 (2)
C2—C1—C14—C9	1.6 (2)	C17—C16—N1—C26	−129.70 (16)
C15—C1—C14—C9	174.79 (14)	C15—C16—N1—C24	−123.73 (19)
C12—C13—C14—C1	179.44 (18)	C17—C16—N1—C24	52.7 (2)
C12—C13—C14—C9	3.0 (3)	N1—C24—N2—C25	0.4 (2)
C8—C9—C14—C1	−1.5 (2)	C26—C25—N2—C24	−0.8 (2)
C10—C9—C14—C1	−179.75 (17)	N3—C25—N2—C24	178.53 (16)
C8—C9—C14—C13	175.20 (17)	C26—C25—N3—O3	179.18 (19)
C10—C9—C14—C13	−3.1 (3)	N2—C25—N3—O3	0.0 (3)
C2—C1—C15—C16	−126.63 (19)	C26—C25—N3—O2	−1.4 (3)
C14—C1—C15—C16	60.0 (2)	N2—C25—N3—O2	179.44 (19)
C1—C15—C16—N1	6.3 (3)		

Symmetry codes: (i) −*x*+1, −*y*+2, −*z*; (ii) *x*+1, *y*+1, *z*; (iii) −*x*+1, −*y*+2, −*z*+1; (iv) −*x*, −*y*+2, −*z*+1.
